# Impact of oral anti–hepatitis B therapy on the survival of patients with hepatocellular carcinoma initially treated with chemoembolization

**DOI:** 10.1186/s40880-015-0017-7

**Published:** 2015-05-14

**Authors:** Zhong-Guo Zhou, Xing-Rong Zheng, Qian Zhou, Ming Shi, Yao-Jun Zhang, Rong-Ping Guo, Yun-Fei Yuan, Min-Shan Chen, Xiao-Jun Lin, Xiang-Ming Lao, Sheng-Ping Li

**Affiliations:** Department of Hepatobiliary Oncology, Sun Yat-sen University Cancer Center; State Key Laboratory of Oncology in South China; Collaborative Innovation Center for Cancer Medicine, Guangzhou, Guangdong 510060 Peoples Republic of China; Department of Infectious Diseases, The Third Affiliated Hospital of Sun Yat-sen University, Guangzhou, Guangdong 510630 Peoples Republic of China; Epidemiology Research Unit, Translational Medicine Research Center, The First Affiliated Hospital of Sun Yat-sen University, Guangzhou, Guangdong 510080 Peoples Republic of China

**Keywords:** Hepatocellular carcinoma, Transcatheter arterial chemoembolization, Hepatitis B virus, Overall survival, Antiviral therapy

## Abstract

**Introduction:**

Most hepatocellular carcinomas (HCC) develop in a background of underlying liver disease including chronic hepatitis B. However, the effect of antiviral therapy on the long-term outcome of patients with hepatitis B virus (HBV)-related HCC treated with chemoembolization is unclear. This study aimed to evaluate the survival benefits of anti-HBV therapy after chemoembolization for patients with HBV-related HCC.

**Methods:**

A total of 224 HCC patients who successfully underwent chemoembolization were identified, and their survival and other relevant clinical data were reviewed. Kaplan-Meier and Cox regression analyses were performed to validate possible effects of antiviral treatment on overall survival (OS).

**Results:**

The median survival time (MST) was 15.9 (95% confidence interval [CI], 9.5–27.7) months in the antiviral group and 9.6 (95% CI, 7.8–13.7) months in the non-antiviral group (log-rank test, *P* = 0.044). Cox multivariate analysis revealed that antiviral treatment was a prognostic factor for OS (*P* = 0.008). Additionally, a further analysis was based on the stratification of the TNM tumor stages. In the subgroup of early stages, MST was significantly longer in the antiviral-treatment group than in the non-antiviral group (61.8 months [95% CI, 34.8 months to beyond the follow-up period] versus 26.2 [95% CI, 14.5–37.7] months, *P* = 0.012). Multivariate analysis identified antiviral treatment as a prognostic factor for OS in the early-stage subgroup (*P* = 0.006). However, in the subgroup of advanced stages, MST of the antiviral-treated group was comparable to that of the non-antiviral group (8.4 [95% CI, 5.2–13.5] months versus 7.4 [95% CI, 5.9–9.3] months, *P* = 0.219). Multivariate analysis did not indicate that antiviral treatment was a significant prognostic factor in this subgroup.

**Conclusion:**

Antiviral treatment is associated with prolonged OS time after chemoembolization for HCC, especially in patients with early-stage tumors.

## Background

Hepatocellular carcinoma (HCC) is the fifth most common cancer in men and the seventh most common in women, and it is the third most common cause of death from cancer worldwide [1]. In 2010, the estimates of new liver cancer cases and deaths were 358,840 and 312,432, respectively, in China [2]. Most HCCs develop based on underlying liver diseases. In Japan, most European countries, and America, approximately 60% of HCC cases are attributed to chronic infection with hepatitis C virus (HCV) [3]. However, in most Asian countries, especially in China, hepatitis B virus (HBV) infection plays the primary role in the etiology of HCC and is frequently observed in HCC patients [4,5]. High levels of serum HBV DNA are associated with an increased risk of developing HCC [6]. Additionally, in HBV-related HCC, high levels of serum HBV DNA appear to be associated with a poor prognosis [7-9]. Thus, careful management of HBV is required in the treatment of hepatic malignancies [10].

Transarterial chemoembolization (TACE) is the most widely used palliative treatment for unresectable HCC; based on clinical evidence, it has shown survival benefits [11,12]. In some studies, TACE has been used as a curative-intent therapy in some selected patients with early-stage HCCs [13-17]. As TACE is a locoregional therapy that is different from systemic chemotherapy, it has the potential to cause HBV reactivation [18,19]. Our previous study revealed that antiviral therapy could reduce the risk of HBV reactivation after TACE [20]. However, the long-term effect of the antiviral treatment on the overall survival (OS) of patients with HCCs undergoing TACE remains unclear, and there are currently few reports concerning this issue.

In this study, we aimed to evaluate the long-term effect of antiviral therapy on OS in a large cohort treated with TACE for HCC.

## Patients and Methods

### Patients and inclusion criteria

This retrospective study was approved by the Institutional Review Board (IRB) at the Sun Yat-sen University Cancer Center. Patients who were initially diagnosed with hepatitis B surface antigen (HBsAg)-positive HCC and received TACE between January 2008 and December 2008 at the Hepatobiliary Department of Sun Yat-sen University Cancer Center were selected for this investigation.

Baseline examinations within 1 week before TACE included serum HBV DNA quantification; detection of HBsAg, hepatitis B surface antibody (HBsAb), hepatitis B core antibody (HBcAb), hepatitis B e antigen (HBeAg), hepatitis B e antibody (HBeAb), and anti-HCV antibody (HCV Ab); serum liver function tests (alanine aminotransferase [ALT], aspartate aminotransferase [AST], alkaline phosphatase, total bilirubin [TBIL], and albumin [ALB]); creatinine levels; prothrombin time (PT); activated partial thromboplastin time (APTT); alpha-fetoprotein (AFP) levels; complete blood counts; and chest radiography. Serum HBV viral loads were measured by using quantitative fluorescence polymerase chain reaction (PCR) detection kit (DaAn Gene Corporation, Guangzhou, China) for HBV DNA with a lower detection limit of 100 IU/mL. The diagnosis of HCC was based on the criteria established by the European Association for the Study of the Liver [21]. Tumor characteristics and TNM stage (Union for International Cancer Control [UICC], 7th version) were evaluated by imaging and/or the intra-operative observation. The HBV DNA status changes after treatment were evaluated within 1 to 5 months after the initial TACE. The changes of HBV DNA levels were grouped into two categories, HBV reactivation and non-reactivation. HBV reactivation was defined as a ≥10-fold increase in the HBV DNA level compared with baseline or the appearance of HBV DNA from an undetectable level at baseline and a post-TACE HBV DNA level >200 IU/mL [19,20,22,23]. The other situations were grouped into non-reactivation.

The inclusion criteria were as follows: a positive result for HBsAg examination, naïve anti-HBV treatment before the current TACE, adequate baseline liver function (Child-Pugh grade A), adequate renal function (serum creatinine < 124 μmol/L), proper baseline Eastern Cooperative Oncology Group performance status (ECOG PS) grades (scores 0–2), and well tolerance of TACE. Exclusion criteria were as follows: a negative result for HBsAg examination, evidence of co-infection with other hepatotropic viruses, human immunodeficiency virus (HIV) infection, any prior treatment for HCC, Child-Pugh grade B or C liver function, any other malignancy, a concurrent non-malignant severe illness, a history of interferon administration, or a history of corticosteroid administration. Specifically, to rule out possible influences caused by the TACE procedure, the patients with severe complications or severe adverse events shortly after TACE (within 1 month) were excluded.

During the study period in our department, there were only 15 patients with Child-Pugh grade B liver function who received an initial treatment of TACE. The number was too small to maintain the homogeneity of the study population; therefore, these 15 patients were excluded.

### Treatments

#### TACE procedure

TACE was performed as described previously [19,24]. When the catheter tip was advanced to the tumor-feeding arteries, one or several chemotherapeutic agents with mixed lipiodol were then slowly injected. Gelatin sponge particles were injected in some sessions if the chemoembolized artery territory did not show stagnant flow. The chemotherapeutic agents included epirubicin (60 mg/session) and/or mitomycin (6 mg/session), carboplatin (300 mg/session), lobaplatin (50 mg/session), and floxuridine (500 mg/session). The selection of various combinations of anticancer agents, lipiodol emulsion dosage, and use of gelform was made on a case-by-case basis. The cycles of TACE were recorded.

#### Subsequent treatment

After the initial TACE, the patients were followed up and received subsequent treatment, including local ablation, hepatectomy, or sorafenib therapy on a case-by-case basis. In the current study, no patients were offered liver transplantation. Follow-up ended on October 1, 2014.

#### Oral antiviral drugs

Of the 224 involved patients, 80 received nucleotide or nucleoside analogs (NUCs) after TACE, and 144 did not. The HBV DNA levels were monitored. Of the 80 patients, 39 received entecavir (ETV) only, and 37 received lamivudine (LAM) only. The other 4 patients initially received LAM treatment. However, because HBV resistance appeared, adefovir dipivoxil (ADV) was added in 2 cases, and LAM was replaced by ETV in the other 2 cases. The choices of LAM and ETV in the current study were based on the availability of agents and the insurance coverage for those agents. The median antiviral time was 13.8 months (range, 1.2 to 82.0 months). No serious adverse events related to antiviral treatment were observed.

### Statistical analysis

Demographic data (mean, standard deviation, median, interquartile [P25, P75], and percentage) were calculated. Analyses were conducted using the independent Student’s *t* test (Mann–Whitney for non-normal distributions), ANOVA, Chi-square test, and Fisher's exact test as appropriate. Kaplan-Meier methods and log-rank tests were used for survival analysis. OS was calculated from the date of initiation of TACE to the date of death or the last follow-up. The Cox proportional hazards model was used in the univariate survival analysis to determine the association of individual clinical variables with OS. All variables with *P* < 0.1, in addition to age and sex, were subsequently subjected to the multivariate Cox regression model to determine the hazards ratios (HRs) and the independence of effects. The proportional hazards assumption was checked by graphic inspection of the linearity of the hazards over time, log-log plots, and plotting Schoenfeld residuals over time. A *P* value less than 0.05 was considered statistically significant. All statistical tests were two-sided. Data were analyzed by using the SAS 9.1 software (SAS Institute, Cary, NC, USA).

## Results

### Study population and baseline clinical characteristics

The clinical characteristics of the study population are shown in Table 1. There were 209 male and 15 female patients with a median age of 49.5 years (range, 16 to 80 years); 53 patients were HBeAg-positive, and 171 were HBeAg-negative. There were 19, 49, and 12 patients who received hepatectomy, local thermal ablation, and sorafenib treatment after TACE, respectively. According to the UICC TNM staging system, there were 46, 25, 68, 63, and 22 patients with stages I, II, IIIa, IIIb + IIIc, and IV HCC, respectively (as there were only 3 patients categorized into stage IIIc, we combined stages IIIc and IIIb).

**Table 1:** **The baseline characteristics of all selected patients with hepatocellular carcinoma (HCC)**

**Characteristic**	**Total (** ***n*** **= 224)**		**Antiviral treatment group (** ***n*** **= 80)**	**Non-antiviral treatment group (** ***n*** **= 144)**	***P*** **value** ^**c**^
**Sex (males:females)**	209:15		78:2	131:13	0.092
**Age (years)** ^**a**^	49.5 (38.5, 58.0)		48.0 (39.5, 56.0)	50.5 (38.0, 59.0)	0.574
**WBC (×10** ^**9**^ **/L)**	6.66 ± 2.12		6.38 ± 1.85	6.82 ± 2.25	0.286
**HBG (g/L)**	137.91 ± 20.28		137.90 ± 22.36	137.90 ± 19.10	0.987
**PLT (×10** ^**9**^ **/L)**	179.91 ± 84.16		170.30 ± 88.10	185.24 ± 81.71	0.076
**ALT (U/L)**	58.38 ± 40.62		65.28 ± 44.99	54.54 ± 37.60	0.081
**AST (U/L)**	84.89 ± 62.44		91.17 ± 63.80	81.40 ± 61.62	0.125
**ALB (g/L)**	40.10 ± 4.40		39.30 ± 4.55	40.54 ± 4.26	0.032
**TBIL (μmol/L)**	16.44 ± 7.61		17.73 ± 8.49	15.72 ± 6.99	0.059
**AFP (ng/mL)** ^**a**^	1301 (50, 38,398)		1436 (53, 37,546)	1210 (50.74, 39,410)	0.820
**AFP (<1,000 ng/mL:>1,000 ng/mL)**	108:116		39:41	69:75	0.905
**PT (s)**	12.58 ± 2.08		12.83 ± 2.05	12.44 ± 2.09	0.052
**APPT (s)**	27.95 ± 3.85		28.22 ± 3.69	27.80 ± 3.94	0.278
**HBeAg (positive:negative)**	53:171		24:56	29:115	0.096
**HBV DNA (log**_**10**_ **IU/mL)**	4.85 ± 1.86		5.50 ± 1.58	4.48 ± 1.92	<0.001
**UICC TNM stage (I:II:IIIa:IIIb + IIIc:IV)**	46:25:68:63:22		9:14:25:26:6	37:11:43:37:16	0.024
**TNM stage (early:advanced)**	71:153		23:57	48:96	0.480
**Cycles of TACE (one:more than one)**	133:91		37:43	96:48	0.003
**Resection after TACE (yes:no)**	19:205		7:73	12:132	0.915
**Local ablation after TACE (yes:no)**	49:175		23:57	26:118	0.064
**Sorafenib therapy after TACE (yes: no)**	12:212		6:74	6:138	0.288
**Subsequent therapy** ^**b**^ **after TACE (yes:no)**	67:157		30:50	37:107	0.065
**Chemotherapeutic agents** **(epirubicin only:>2 agents)**	53:171		18:62	35:109	0.761

^**a**^The values are presented as median followed by interquartile (P25 and P75) in the parentheses. ^**b**^Subsequent therapy means any treatment after TACE, including resection, local ablation, or sorafenib therapy. ^**c**^Differences between the antivirus group and the non-antivirus group. WBC, white blood cells; HBG, hemoglobin; PLT, platelets; ALT, alanine aminotransferase; AST, aspartate aminotransferase; ALB, serum albumin; TBIL, total bilirubin; AFP, α-fetoprotein; PT, prothrombin time; APTT, activated partial thromboplastin time; HBeAg, hepatitis B e antigen; HBV, hepatitis B virus; UICC TNM, International Union Against Cancer tumor-node-metastasis; TACE, transcatheter arterial chemoembolization.

The mean baseline HBV DNA level in the antiviral treatment group was significantly higher than that in the non-antiviral treatment group (5.50 ± 1.58 log_10_IU/mL versus 4.48 ± 1.92 log_10_IU/mL, *P* < 0.01), which may have been because antiviral treatment was selectively used for patients with high viral loads. There were 53.8% (43 of 80) of patients undergoing more than one session of TACE in antiviral treatment group, which was significantly higher than the percentage in the non-antiviral treatment group (33.3%, 48 of 144) (*P* = 0.003). The frequency of local thermal therapy after TACE in the antiviral treatment group was 28.8% (23 of 80), higher than that in the non-antiviral treatment group (18.1%, 26 of 144) (*P* = 0.064). One to 5 months after the initial TACE, there were 2 of 80 cases (2.5%) of HBV reactivation in the antiviral therapy group and 20 of 144 (13.9%) in the non-antiviral therapy group (*P* < 0.001).

The median follow-up period for all patients was 9.9 months (range, 1.3 to 82.0 months). The median survival time was 11.0 months (95% CI, 9.2 to 15.9 months). A total of 177 patients died, including 59 patients in antiviral treatment group and 118 patients in non-antiviral treatment group. The 0.5-, 1-, 2-, 3-, 4-, 5-, and 6-year OS rates for all patients were 69.7%, 47.9%, 32.3%, 23.2%, 20.1%, 16.9%, and 14.4%, respectively.

### Univariate and multivariate analyses of factors contributing to OS

The univariate analysis identified that the following factors were significantly associated with OS time for all cases: age, sex, TNM stage, AST, ALB, TBIL, AFP, antiviral treatment, subsequent local ablation, and resection. The multivariate analysis revealed that sex, TNM stage, ALB, antiviral treatment, local ablation, and resection were significant prognostic factors linked to OS time (Table 2).

**Table 2:** **The relationship between clinical characteristics and overall survival in 224 patients: Cox’s regression analysis**

**Variable**	**Univariate analysis**		**Multivariate analysis**			
	***P*** **value**		**Hazard ratio**	**95% CI**	***P*** **value**	
**Age**	0.013		0.988	0.975–1.002	0.084	
**Sex**	0.020		2.424	1.139–5.156	0.022	
**HBV DNA (log**_**10**_ **IU/mL)**	0.602		–	–	–	
**ALT**	0.607		–	–	–	
**TNM stage (early:advanced)**	<0.001		2.031	1.410–2.925	<0.001	
**Cycles of TACE**	0.346		–	–	–	
**AST**	<0.001		1.001	0.998–1.005	0.496	
**ALB**	0.043		0.950	0.913–0.988	0.010	
**TBIL**	0.028		1.007	0.986–1.029	0.511	
**AFP**	<0.001		1.400	0.979–2.003	0.066	
**APTT**	0.259		–	–	–	
**Antiviral treatment**	0.044		0.635	0.454–0.888	0.008	
**Local ablation**	<0.001		0.241	0.133–0.436	<0.001	
**Resection**	<0.001		0.298	0.148–0.599	0.001	
**HBeAg**	0.977		–	–	–	
**PT**	0.746		–	–	–	
**Sorafenib therapy**	0.825		–	–	–	
**Chemotherapeutic agents (epirubicin only:>2 agents)**	0.798		–	–	–	

CI, confidence interval. Other abbreviations as in Table 1.

**Figure 1:**
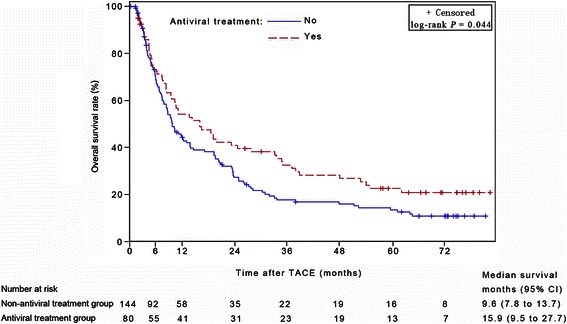
Kaplan-Meier curves of overall survival for 224 hepatocellular carcinoma (HCC) patients with or without antiviral treatment. Survival rate in the antiviral treatment group was significantly higher than that in the non-antiviral treatment group. TACE, transarterial chemoembolization; CI, confidence interval.

The median survival times in the antiviral treatment group and the non-antiviral treatment group were 15.9 months (95% CI, 9.5 to 27.7 months) and 9.6 months (95% CI, 7.8 to 13.7 months), respectively. Survival time was significantly longer in the antiviral treatment group than in the non-antiviral treatment group (*P* = 0.044) (Figure 1).

**Figure 2:**
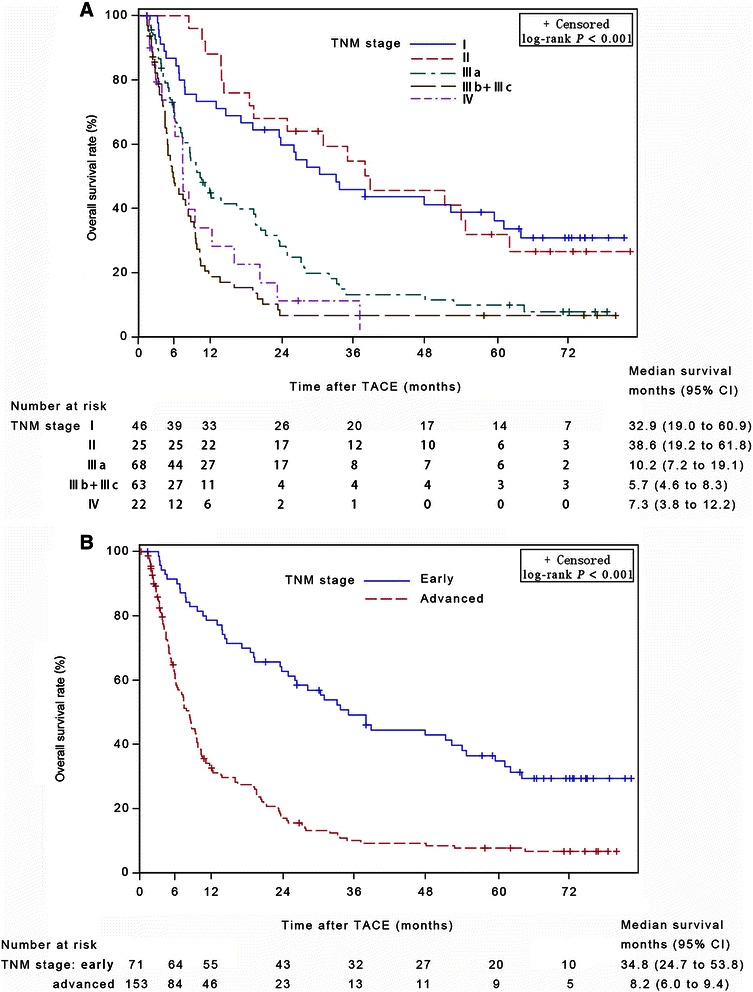
Kaplan-Meier curves of overall survival for 224 patients with HCC at different stages. **A**, the tumor stages were categorized into TNM stages I, II, IIIa, IIIb + IIIc, and IV. **B**, the tumor stages were categorized into TNM early stage (including stages I and II) and advanced stage (including stages III and IV). TACE, transarterial chemoembolization; CI, confidence interval.

The tumor stage also significantly affected the OS time. Figure 2A shows that TNM stage was significantly associated with OS time. When we categorized TNM stage into two main types, early stage (stages I and II) and advanced stage (stages III and IV), the tendency was still obvious and significant (Figure 2B). The median survival times were 34.8 (95% CI, 24.7–53.8) months for patients with early-stage diseases and 8.2 (95% CI, 6.0–9.4) months for patients with advanced-stage diseases (*P* < 0.001).

### Stratification analysis based on the UICC TNM staging

#### TNM early stage

**Figure 3:**
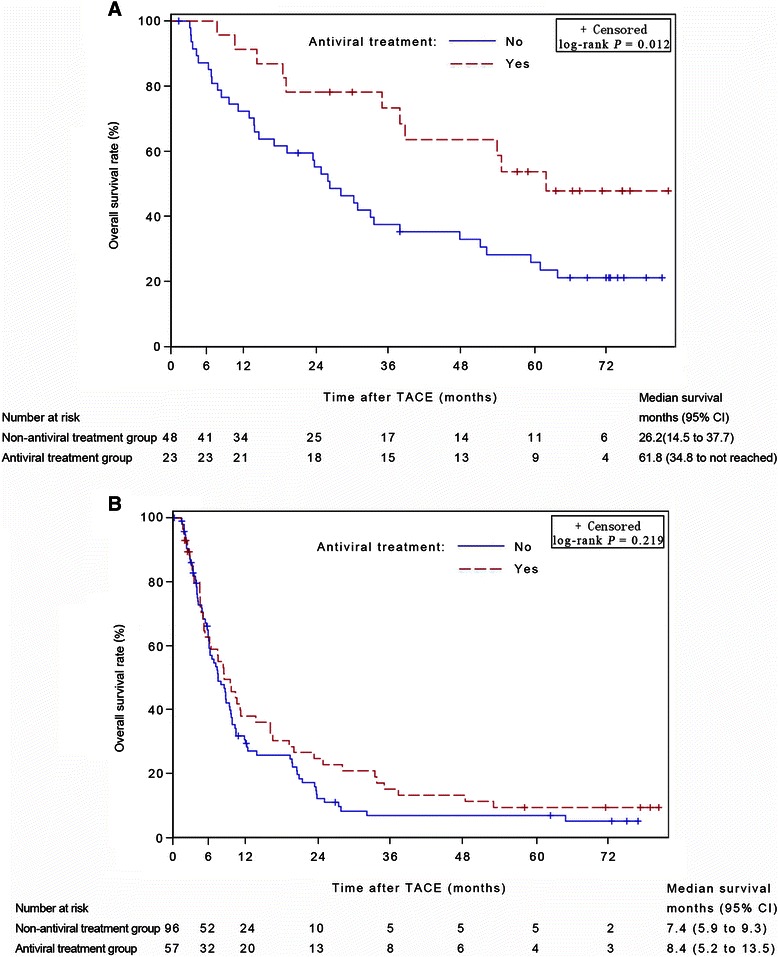
Kaplan-Meier subgroup analysis stratified according to TNM stage. **A**, for the 71 patients with TNM early-stage diseases (including stages I and II), the survival rate in the antiviral treatment group was significantly higher than that in the non-antiviral treatment group. **B**, for the 153 patients with TNM advanced stage diseases (including stages III and IV), there was no significant difference between the antiviral and non-antiviral treatment groups. TACE, transarterial chemoembolization; CI, confidence interval.

The characteristics of 71 patients with early-stage diseases are summarized in Table 3. The median survival time for the antiviral treatment group was 61.8 months (95% CI, 34.8 months to beyond the follow-up period), and 26.2 months (95% CI, 14.5–37.7 months) for the non-antiviral group (log-rank test, *P* = 0.012). Antiviral therapy was associated with significantly longer survival time compared with non-antiviral therapy in HCC patients with early-stage diseases who underwent TACE (Figure 3A).

**Table 3:** **The baseline characteristics of 224 patients with HCC based on the stratification with the TNM staging system**

**Characteristic**		**TNM early stage**					**TNM advanced stage**			
		**Total (** ***n*** ** = 71)**	**Antiviral treatment group (** ***n*** ** = 23)**	**Non-antiviral group (** ***n*** ** = 48)**	***P*** **value**		**Total (** ***n*** ** = 153)**	**Anti-viral group (** ***n*** ** = 57)**	**Non-antiviral group (** ***n*** ** = 96)**	***P*** **value**
**Sex (males:females)**		108:11663:8	22:1	41:7	0.261		146:7	56:1	90:6	0.198
**Age** ^**a**^ **(years)**		54.5 (45.0, 62.0)	55.0 (48.0, 62.0)	52.5 (42.0, 62.5)	0.690		46.0 (36.5, 57.0)	45.0 (37.0, 53.0)	48.0 (36.0, 57.0)	0.427
**WBC (×10** ^**9**^ **/L)**		6.29 ± 1.91	6.38 ± 1.94	6.25 ± 1.91	0.649		6.84 ± 2.20	6.38 ± 1.83	7.11 ± 2.35	0.090
**HBG (g/L)**		139.02 ± 19.74	140.77 ± 17.61	138.18 ± 20.81	0.609		137.39 ± 20.57	136.79 ± 24.06	137.74 ± 18.30	0.783
**PLT (×10** ^**9**^ **/L)**		153.37 ± 79.37	147.78 ± 77.98	156.04 ± 80.70	0.746		192.22 ± 83.71	179.39 ± 90.93	199.84 ± 78.62	0.052
**ALT (U/L)**		50.19 ± 32.14	55.11 ± 28.20	47.83 ± 33.89	0.136		62.18 ± 43.58	69.38 ± 49.83	57.90 ± 39.06	0.289
**AST (U/L)**		65.63 ± 44.32	60.38 ± 29.26	68.14 ± 50.05	0.883		93.83 ± 67.54	103.59 ± 69.70	88.03 ± 65.90	0.092
**ALB (g/L)**		40.14 ± 4.59	40.40 ± 4.82	40.02 ± 4.52	0.976		40.08 ± 4.32	38.86 ± 4.41	40.80 ± 4.13	0.009
**TBIL (μmol/L)**		15.72 ± 6.68	16.09 ± 6.76	15.54 ± 6.71	0.811		16.77 ± 8.00	18.40 ± 9.07	15.81 ± 7.16	0.056
**AFP** ^**a**^ **(ng/mL)**		197.70 (7.72, 3065)	63.68 (5.12, 804.7)	650 (9.33, 9419.50)	0.074		4682 (202.80, 76308)	7097 (363.6, 85094)	4571 (106.58, 67557.50)	0.522
**AFP (<1,000 ng/mL:>1,000 ng/mL)**		47:24	19:4	28:20	0.043		61:92	20:37	41:55	0.352
**PT (s)**		12.46 ± 2.34	12.77 ± 2.47	12.31 ± 2.28	0.373		12.63 ± 1.96	12.85 ± 1.88	12.50 ± 2.00	0.103
**APPT (s)**		27.66 ± 3.76	27.57 ± 4.35	27.71 ± 3.50	0.749		28.08 ± 3.89	28.48 ± 3.39	27.85 ± 4.16	0.127
**HBeAg positive:negative**		15:56	7:16	41:78:40	0.184		38:115	17:40	21:75	0.271
**HBV DNA (log**_**10**_ **IU/mL)**		4.58 ± 1.93	5.24 ± 1.97	4.26 ± 1.84	0.033		4.97 ± 1.83	5.60 ± 1.39	4.59 ± 1.95	0.001
**Cycles of TACE (one:more than one)**		43:28	8:15	35:13	0.002		90:63	29:28	61:35	0.124
**Resection after TACE (yes:no)**		11:60	3:20	8:40	1.000		8:145	4:53	5:92	0.471
**Local therapy after TACE (yes:no)**		29:42	12:11	17:31	0.179		20:133	11:46	9:87	0.078
**Sorafenib use after TACE (yes:no)**		5:66	3:20	2:46	0.320		7:146	3:54	4:92	0.712
**Subsequent therapy (yes:no)**		37:34	15:8	22: 26	0.126		30:123	15:42	15:81	0.107
**Chemotherapeutic agents (epirubicin only: >2 agents)**		19:52	5:18	14:34	0.508		34:119	13:44	21:75	0.893

^**a**^The values are presented as median followed by interquartile (P25 and P75) in the parentheses. Abbreviations as in Table 1.

Univariate analysis identified that the following factors were significantly associated with OS for the TNM early-stage patients: AST, AFP, antiviral treatment, local therapy, and resection. Multivariate analysis revealed that sex, antiviral treatment, local ablation, and resection were significant prognostic factors associated with OS (Table 4).

**Table 4:** **The relationship between clinical characteristics and overall survival in HCC patients based on the stratification with the TNM staging system: Cox’s regression analysis**

**Variable**		**TNM early stage (** ***n*** ** = 71)**						**TNM advanced stage (** ***n*** ** = 153)**				
		**Univariate analysis**		**Multivariate analysis**				**Univariate analysis**		**Multivariate analysis**		
		***P*** **value**		**Hazard ratio**	**95% CI**	***P*** **value**		***P*** **value**		**Hazard ratio**	**95% CI**	***P*** **value**
**Age**		0.821		1.001	0.978–1.025	0.920		0.075		0.975	0.956–0.995	0.016
**Sex**		0.158		3.946	1.332–11.690	0.013		0.372		1.891	0.641–5.577	0.248
**HBV DNA (log**_**10**_ **IU/mL)**		0.408		–	–	–		0.328		–	–	–
**ALT**		0.445		–	–	–		0.994		–	–	–
**Cycles of TACE**		0.885		–	–	–		0.070		1.305	0.836–2.037	0.242
**AST**		0.016		1.004	0.996–1.012	0.363		0.001		1.002	0.999–1.005	0.201
**ALB**		0.149		–	–	–		0.066		0.945	0.893–1.000	0.051
**TBIL**		0.711		–	–	–		0.024		1.008	0.982–1.035	0.555
**AFP**		0.022		2.087	0.920–4.736	0.078		0.067		1.144	0.741–1.767	0.545
**APTT**		0.709		–	–	–		0.068		0.988	0.942–1.037	0.633
**Antiviral treatment**		0.012		0.372	0.183–0.756	0.006		0.219		–	–	–
**Local ablation**		0.005		0.280	0.136–0.577	0.001		<0.001		0.211	0.095–0.472	<0.001
**Resection**		0.023		0.152	0.049–0.473	0.001		0.008		0.308	0.118–0.801	0.016
**HBeAg**		0.958		–	–	–		0.621		–	–	–
**PT**		0.241		–	–	–		0.765		–	–	–
**Sorafenib therapy**		0.943		–	–	–		0.782		–	–	–
**Chemotherapeutic agents** **(epirubicin only: >2 agents)**		0.211		–	–	–		0.073		0.881	0.540–1.439	0.613

Abbreviations as in Table 1.

#### TNM advanced stage

The characteristics of 153 patients with advanced-stage diseases are summarized in Table 3. The Kaplan-Meier analysis revealed that there was no significant difference in OS between the antiviral treatment and the non-antiviral treatment groups in the patients with advanced-stage diseases (8.4 [95% CI, 5.2–13.5] months versus 7.4 [95% CI, 5.9–9.3] months, *P* = 0.219 by log-rank test) (Figure 3B).

Univariate analysis identified that AST, TBIL, local ablation, and resection were significantly associated with OS for the patients with advanced-stage diseases. Multivariate analysis revealed that age, local ablation, and resection were significant prognostic factors associated with OS (Table 4).

## Discussion

Only limited amount of data are available on the prognostic value of antiviral therapy in HCC patients undergoing TACE. Recently, the impact of antiviral therapy on the survival after radical resection for HBV-related HCC has been reported [25-28]. Most studies have indicated that antiviral treatment can improve the prognosis after surgery, including the increase of disease-free survival and OS rates [25,28]. However, there is little information about the effect of antiviral therapy on the long-term outcome of HCC patients undergoing TACE.

For all of the cases in this study, patients with antiviral treatment had significantly prolonged OS time compared with those without antiviral treatment. The Cox multivariate analysis demonstrated that antiviral therapy was an independent predictor for the OS time of HCC patients who underwent TACE. The results showed that antiviral treatment conferred a survival benefit in patients with HBV-related HCC undergoing TACE.

Although not fully clarified [29], the role played by antiviral therapy in prolonging the survival time may include several aspects [28,30]. First, antiviral therapy can decrease HBV replication and levels of HBV DNA, which are regarded as risk factors for the development of HCC [6]. In a study evaluating the relationship between HBV DNA levels and the survival of patients with HCC treated by TACE, a high pre-TACE serum level of HBV DNA was observed to be associated with poor OS and rapid progression of HCC after TACE [31]. The cause of deaths was not hepatitis exacerbation but cancer progression. Second, the incidence of HBV reactivation and the subsequent hepatitis can be decreased, reducing the impairment of liver function and possibly rendering patients to better tolerate further HCC treatment. In the current study, 1 to 5 months after the initial TACE, there were 2 of 80 cases (2.5%) of HBV reactivation in the antiviral therapy group and 20 of 144 (13.9%) in the non-antiviral therapy group (*P* < 0.001), which is similar to the results of our previous study [19,20]. In addition, 53.8% (43 of 80) of the patients in the antiviral treatment group received more than one session of TACE and 28.8% (23 of 80) of the patients received post-TACE local ablation, which were higher than the rates in the non-antiviral treatment group (Table 1). Because the tumor stages and the baseline liver function were comparable between the groups, it is reasonable to postulate that the better hepatic reserves in the antiviral therapy patients accounted for the higher rate of multiple sessions of TACE or local ablation for HCC. This result is consistent with those reported by Chan *et al*. [26] and Toyoda *et al*. [32]. Third, although not well defined, antiviral therapy may be associated with a reduced risk of late-phase recurrence of HCC and therefore increase OS rate. Although most patients in the current study had intermediate- or advanced-stage diseases (Table 1), multiple TACE sessions with subsequent local ablation or resection may achieve favorable or even curable effects in some selected cases [33].

In the current study, all patients had Child-Pugh grade A liver function and ECOG PS grades 0–2. Because the liver functional status and the physical status of patients in this study were similar, we used the TNM staging system in our analysis instead of the Barcelona Clinic Liver Cancer (BCLC) staging system, in a manner similar to that used by Chan *et al*. [26], who evaluated the effect of antiviral treatment on the prognosis of HCC patients undergoing major hepatectomy. For early-stage HCC, surgical resection or radiofrequency ablation (RFA) are regarded as first-line therapies, and liver transplantation is an alternative. TACE is generally recommended for intermediate- or advanced-stage diseases in most clinical guidelines [34]. Actually, the majority of patients with early-stage HCCs in our institute have been treated with hepatectomy or RFA. However, some patients had contraindications to surgery or RFA, including tumor location unsuitable for resection or ablation [14], and others chose TACE after considering all the options [13]. Furthermore, in the current study, we chose UICC TNM staging (7th edition), which does not use the diameter of the solitary tumor as a criterion in the classification of T1 and T2 lesions. Thus, very large but solitary tumors, which were unsuitable for resection, were categorized as early-stage diseases. In early tumor stages (TNM stages I and II), the median survival time for patients with antiviral treatment was significantly longer than that of those with non-antiviral therapy (Figure 3A). The Cox multivariate analysis revealed that antiviral treatment was an independent factor for OS in patients with early-stage tumors (Table 4). In patients with advanced-stage HCCs (TNM stages III and IV), there was no significant difference in OS between the antiviral and the non-antiviral treatment groups (Figure 3B), and the Cox multivariate analysis did not identify antiviral treatment as a predictor for OS (Table 4). These findings were somewhat similar to those reported by Chan *et al*. [26], who evaluated the effect of antiviral treatment on the prognosis of HCC patients undergoing major hepatectomy. They stratified the patients according to the American Joint Committee on Cancer (AJCC) TNM stage and the status of major vascular invasion. The authors found that overall and disease-free survival rates were increased after antiviral treatment in patients with AJCC TNM stages I and II tumors or in those without major vascular invasion, whereas no survival benefit was observed for patients with stage III tumors or with major vascular invasion. We postulated that the main reason for the discrepancy in the results in different subgroups is that the long-term effect of NUCs has not yet been determined in patients with advanced-stage tumors who had short survival times. In the current study, the median survival time was shorter in patients with advanced-stage diseases than in those with early-stage diseases (8.2 months versus 34.8 months), which may help explain how the effect of antiviral therapy on survival differs depending on the HCC stage. The results indicate that it is much more important to begin antiviral treatment in HCC patients with early-stage tumors who are expected to have longer survival time compared with those with advanced-stage tumors.

Although the antiviral treatment did not show a survival benefit in patients with advanced-stage diseases in the current study, that should not be interpreted as indicating that there is no need for antiviral therapy in all advanced-stage patients undergoing TACE. The current study only included patients who had Child-Pugh grade A liver function. Patients with severe post-TACE complications (including severe hepatitis or liver failure) were excluded from this study. A prospective randomized study demonstrated that preemptive lamivudine therapy could effectively reduce the risk of hepatitis caused by HBV reactivation and hepatic morbidity during TACE [35]. Our previous study also revealed that anti-HBV therapy can reduce the risk of HBV reactivation, thus reducing the risk of liver failure in patients undergoing TACE [20]. Therefore, we still recommend the close monitoring of the patients with advanced-stage HCC after TACE and the timely use of NUCs in the patients with poor baseline liver function, the presence of cirrhosis, high levels of HBV replication, and/or HBV reactivation [18,20,36].

It should be noted that subsequent local thermal ablation and/or subsequent resection after TACE was also a positive contributing factor to OS. Nearly 30% (67 of 224) of the patients received other types of treatment after TACE. Tables 1 and 3 show the fractions of patients who underwent subsequent therapies. Patients with early-stage HCCs received more subsequent therapies than those with advanced-stage diseases; 15.5% (11 of 71) of the patients with early-stage HCCs underwent resection after TACE, whereas in the patients with advanced-stage diseases, the percentage was only 5.2% (8 of 153); 40.8% (29 of 71) of the patients with early-stage HCCs underwent RFA after TACE, whereas in the patients with advanced-stage diseases, the percentage was only 13.1% (20 of 153). The fractions of each subsequent therapy in the antiviral and the non-antiviral groups were not significantly different (*P* > 0.05, Tables 1 and 3). In addition, sex was also a risk factor for survival in HCC patients in the current study, which is similar to previous findings [37].

In this retrospective study, to eliminate the possible adverse effects caused by treatment-related morbidity or mortality shortly after TACE, we only chose the patients with normal liver function before TACE (Child-Pugh grade A) and excluded those with severe complications shortly after TACE. Thus, we only evaluated the patients who were likely to tolerate the TACE procedures well. Therefore, the conclusions may not be generalizable to all HCC patients undergoing TACE, including those who cannot tolerate the chemoembolization well. Administration of NUCs was not randomized into the two groups in this retrospective cohort study, which may have caused selection bias. Thus, well-designed prospective randomized controlled trials are needed to confirm these findings.

In summary, our results demonstrated that antiviral treatment was associated with prolonged OS time after TACE for treatment of HBV-related HCC, especially in relatively early-stage HCC.
